# Coupled Bone–Muscle Degeneration in Chronic Pancreatitis: A Juvenile Porcine Model of Secondary Osteosarcopenia

**DOI:** 10.3390/ijms26167690

**Published:** 2025-08-08

**Authors:** Siemowit Muszyński, Michał Świetlicki, Dorota Wojtysiak, Agnieszka Grzegorzewska, Piotr Dobrowolski, Małgorzata Świątkiewicz, Marcin B. Arciszewski, Iwona Puzio, Joanna Bonior, Agnieszka Tomczyk-Warunek, Maria Mielnik-Błaszczak, Ewa Tomaszewska

**Affiliations:** 1 Department of Biophysics, University of Life Sciences in Lublin, 20-950 Lublin, Poland; siemowit.muszynski@up.lublin.pl; 2 Department of Applied Physics, Lublin University of Technology, 20-618 Lublin, Poland; m.swietlicki@pollub.pl; 3 Department of Animal Genetics, Breeding and Ethology, University of Agriculture in Kraków, 30-059 Kraków, Poland; dorota.wojtysiak@urk.edu.pl; 4 Department of Animal Physiology and Endocrinology, University of Agriculture in Kraków, 30-059 Kraków, Poland; agnieszka.grzegorzewska@urk.edu.pl; 5 Department of Functional Anatomy and Cytobiology, Maria Curie Sklodowska University, 20-033 Lublin, Poland; piotr.dobrowolski@umcs.lublin.pl; 6 Department of Animal Nutrition and Feed Science, National Research Institute of Animal Production, 32-083 Balice, Poland; malgorzata.swiatkiewicz@iz.edu.pl; 7 Department of Animal Anatomy and Histology, University of Life Sciences in Lublin, 20-950 Lublin, Poland; mb.arciszewski@wp.pl; 8 Department of Animal Physiology, University of Life Sciences in Lublin, 20-950 Lublin, Poland; iwona.puzio@up.lublin.pl; 9 Faculty of Medicine and Health Sciences, University of Applied Sciences in Nowy Sącz, 33-300 Nowy Sącz, Poland; jbonior@ans-ns.edu.pl; 10 Department of Traumatology, Orthopedics and Rehabilitation, Medical University of Lublin, 20-954 Lublin, Poland; agnieszka.tomczyk-warunek@umlub.pl; 11 Chair and Department of Developmental Dentistry, Medical University of Lublin, 20-093 Lublin, Poland; mielnikmb@gmail.com

**Keywords:** bone strength, muscle fibers, osteosarcopenia, porcine model

## Abstract

Osteosarcopenia, characterized by concurrent bone loss and muscle wasting, significantly impacts mobility and quality of life. While age-related primary osteosarcopenia is well-studied, secondary osteosarcopenia (SOS) caused by chronic diseases remains poorly understood, particularly in young individuals. The present study aimed to comprehensively characterize musculoskeletal alterations associated with SOS using a juvenile porcine model of cerulein-induced chronic pancreatitis. Femoral bone analysis included densitometry, mechanical testing, histomorphometry, and serum bone turnover markers. The quadriceps femoris muscle was evaluated through histological analysis and gene expression profiling of antioxidant enzymes and apoptotic regulators. Animals with SOS showed significantly reduced femoral BMD compared to controls, with altered cortical geometry and compromised mechanical properties. Trabecular bone analysis revealed classic osteoporotic changes with decreased bone volume fraction. Negative changes were also observed in the growth plate morphology, indicating impaired endochondral ossification. Bone turnover markers indicated elevated bone resorption and altered formation. Muscle analysis demonstrated sarcopenic changes with selective atrophy of fast-twitch type II fibers and increased fiber density. At the molecular level, SOS muscles exhibited downregulated expression of *CAT* and *CASP3*, suggesting muscle atrophy predominantly mediated by oxidative stress and caspase-independent proteolysis rather than classical apoptosis. In conclusion, chronic pancreatitis in young pigs induces coupled bone and muscle degeneration consistent with secondary osteosarcopenia, demonstrating that muscle–bone crosstalk dysfunction occurs early in chronic inflammatory disease.

## 1. Introduction

Bones and muscles operate as a functional unit in both mechanical and endocrine capacities; to some extent, they regulate each other via numerous signaling factors acting through endocrine and paracrine pathways [1,2]. Consequently, sarcopenia, defined by losses in body mass, muscle strength, and muscle mass, and osteopenia/osteoporosis, a systemic metabolic bone disease marked by compromised microarchitecture and increased fragility, share common mechanisms. Both conditions impair mobility and quality of life, and they can coexist with diseases that are not strictly age-related. During aging or disease, muscle atrophy accelerates trabecular bone loss, while declining bone strength promotes further muscle wasting and functional decline, contributing to osteoporosis [3]. An epidemiological study of 17,891 adults (18–97 years) found that sarcopenic individuals were twice as likely to have osteoporosis compared to healthy individuals [4].

As both osteoporosis and sarcopenia share common causes, they often co-exist in osteosarcopenia [5,6], which combines muscle weakness with increased bone fragility due to bone loss and decreased bone mineral density (BMD). Osteosarcopenia is a key factor in both (fragility) fractures and stress (fatigue) fractures of the hips and ribs [6,7,8]. While experimental studies and clinical statistics have provided insight into age-related primary osteosarcopenia, secondary disease-related osteosarcopenia (SOS) remains poorly characterized due to its multifactorial nature involving cancer, chronic organ failure, immobilization, or obesity. SOS is considered more prevalent than primary osteosarcopenia, with younger individuals more likely to suffer from SOS [9,10].

Secondary osteosarcopenia is often triggered by chronic diseases, including pancreatitis, a significant gastroenterological issue associated with severe complications and high mortality [11,12,13,14]. Chronic pancreatitis (CP), a progressive inflammatory disease, is difficult to diagnose due to its long asymptomatic phase, during which both exocrine and endocrine pancreatic functions are gradually impaired. CP presents with symptoms varying by disease severity, commonly leading to weight loss and multi-organ dysfunction. Approximately 17% of patients with CP exhibit SOS [11,14,15]. CP is typically diagnosed after the pancreas has sustained over 90% damage, leading to severe systemic weakening where sarcopenic changes become prominent. The development of osteoporosis in such cases is often overlooked due to focus on the primary disease [16,17]. Osteoporotic changes in SOS are underdiagnosed and difficult to monitor [18]. Because obtaining bone tissue samples during inflammatory processes in humans is challenging, our understanding of SOS and CP pathogenesis relies largely on experimental animal models that focus on older populations, leaving research on youth-onset sarcopenia in its early stages.

The lack of studies on SOS in young individuals, including animal models, means that our understanding of these processes remains limited. This is particularly important regarding bone tissue, whose specific characteristics prevent simple extrapolation from studies conducted on elderly individuals with primary osteosarcopenia.

The aim of this work is to identify, classify, and determine the extent of pathophysiological changes in bone and muscle metabolism in young individuals with SOS caused by chronic inflammatory disease. We hypothesize that experimental CP in young animals will lead to bone loss associated with muscle changes. Changes in femur mineralization, mechanical strength, histomorphometric parameters of trabecular bone, and serum bone turnover markers will be analyzed to assess osteoporosis. For sarcopenia, quadriceps femoris muscle histology and expression of genes encoding antioxidant proteins and those involved in programmed cell death will be examined.

## 2. Results

As reported previously [19], the body weight of all the growing pigs increased significantly over the entire seven-week experimental period; however, SOS pigs weighed significantly less than control pigs at weeks 6 and 7, corresponding to the week prior to and at the time of euthanasia. Additionally, serum concentrations of anabolic (oxytocin) and catabolic (myostatin) mediators exhibited alterations typical of muscle atrophy; oxytocin levels were markedly reduced, whereas myostatin levels were markedly elevated from the third week post-induction of pancreatitis until euthanasia [20]. Ultrasound analysis revealed a decrease in muscle mass, including load-bearing quadriceps femoris [20].

### 2.1. Bone Analysis

#### 2.1.1. Femoral Morphometry and Densitometry

**Figure 1:**
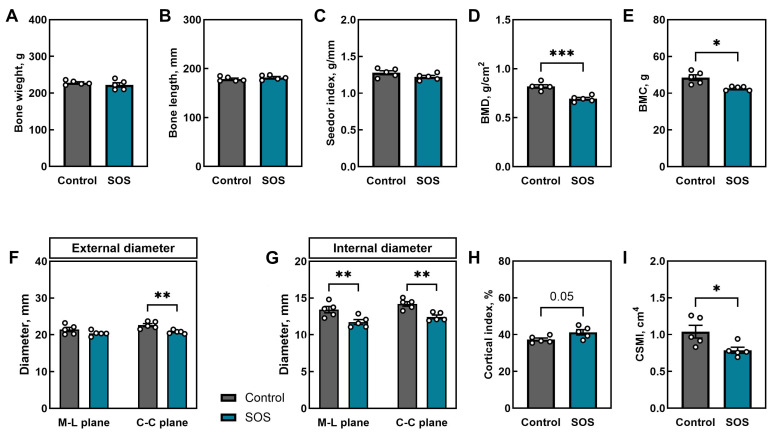
Femoral morphometry, densitometry, and mid-diaphyseal geometry in control and SOS pigs: (**A**) bone weight, (**B**) bone length, (**C**) Seedor index, (**D**) bone mineral density (BMD), (**E**) bone mineral content (BMC), bone mid-diaphysis (**F**) external diameters and (**G**) internal diameters in mediolateral (M–L) and craniocaudal (C–C) planes, (**H**) cortical index, and (**I**) cross-sectional moment of inertia (CSMI). Data are mean ± SEM with individual values shown (n = 5 per group). Statistical significance: * *p* < 0.05, ** *p* < 0.01, *** *p* < 0.001, trend: *p* = 0.05.

There were no differences in femur weight or length (Figure 1A,B); consequently, the Seedor index, an indicator of bulk density, was also unchanged (Figure 1C). However, femora from SOS pigs exhibited significantly lower BMD (*p* < 0.005; Figure 1D) and BMC (*p* < 0.01; Figure 1E). Using the control group as a reference, the standardized BMD index for the SOS group was −3.2 (95% CI_boot_: −4.17 to −2.20). Although this result should be interpreted cautiously, it indicates a substantial reduction in BMD in the SOS group. Despite no change in femur length, bone mid-diaphysis geometry was altered; SOS femora showed a reduced external diameter in the craniocaudal plane (*p* < 0.01; Figure 1F) and reduced internal diameters in both mediolateral and craniocaudal planes (*p* < 0.01 for both; Figure 1G). Consequently, there was a trend toward an increased cortical index at the mid-diaphysis cross-section (*p* = 0.05; Figure 1H) and a significantly reduced cross-sectional moment of inertia (CSMI) (*p* < 0.05; Figure 1I). The detailed results of analysis (means, 95% CI, and *p*-values) are presented in supplementary data (Table S1).

#### 2.1.2. Mechanical Properties of Femora

**Figure 2:**
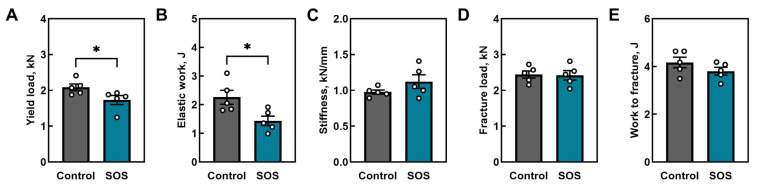
Mechanical properties of femora in control and SOS pigs: (**A**) yield load, (**B**) elastic work (energy absorbed to yield), (**C**) stiffness, (**D**) fracture load, (**E**) work to fracture. Data are mean ± SEM with individual values shown (n = 5 per group). Statistical significance: * *p* < 0.05.

Mechanical testing by three-point bending revealed several alterations in the femora of SOS pigs (Figure 2). Yield load and elastic work (energy absorbed up to yield) were both significantly reduced in the SOS group (*p* < 0.05 for both; Figure 2A,B), whereas stiffness remained unchanged (Figure 2C). On the other hand, fracture load (Figure 2D) and work to fracture (Figure 2E) did not differ between groups, indicting no structural changes in the elastic region of bone deformation. The detailed results of the analysis are presented in the supplementary data (Table S2).

#### 2.1.3. Trabecular Bone Microarchitecture and Growth Plate Morphology

**Figure 3:**
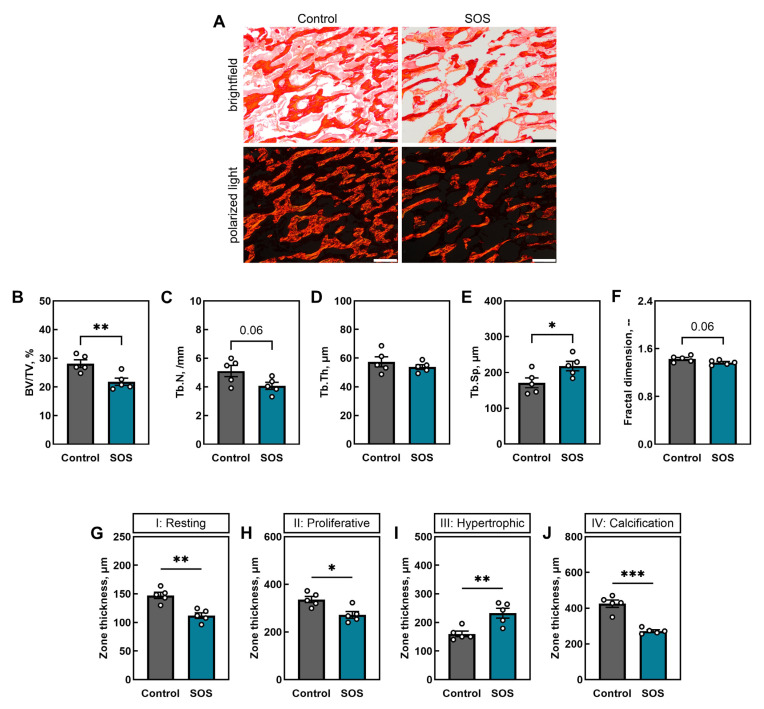
Trabecular bone microarchitecture and growth plate morphology in distal femora of control and SOS pigs. (**A**) Representative picrosirius-red-stained sections of distal femoral metaphysis trabeculae in brightfield and polarized light. Quantitative analysis of trabecular parameters: (**B**) bone volume fraction (BV/TV), (**C**) trabecular number (Tb.N), (**D**) trabecular thickness (Tb.Th), (**E**) trabecular separation (Tb.Sp), and (**F**) fractal dimension. Growth plate zone thicknesses: (**G**) resting zone (I), (**H**) proliferative zone (II), (**I**) hypertrophic zone (III), and (**J**) calcification zone (IV). All the scale bars show 400 μm. Data are mean ± SEM with individual values shown (n = 5 per group). Statistical significance: * *p* < 0.05, ** *p* < 0.01, *** *p* < 0.001; trend: *p* = 0.06.

Figure 3A shows representative PSR-stained sections of trabecular bone from the distal femoral metaphysis in both groups, imaged in brightfield and corresponding polarized light, demonstrating that trabecular microarchitecture was markedly compromised in the femora of SOS pigs. Quantitative analysis revealed that bone volume fraction (BV/TV) was significantly reduced in the SOS group (*p* < 0.01; Figure 3B), and the trabecular number (Tb.N) tended to be lower (*p* = 0.06; Figure 3C), while trabecular thickness (Tb.Th) remained unchanged (Figure 3D). Trabecular separation was significantly increased in SOS femora (*p* < 0.05; Figure 3E), and fractal dimension showed a downward trend (*p* = 0.06; Figure 3F).

Assessment of growth plate morphology revealed alterations in chondrocyte zones (Figure 3G–J). The resting zone (I) thickness was significantly reduced in the femora of the SOS pigs (*p* < 0.01; Figure 3G), as was the proliferative zone (II) (*p* < 0.05; Figure 3H). In contrast, the hypertrophic zone (III) was significantly expanded in the femora of SOS animals (*p* < 0.01; Figure 3I), whereas the calcification zone (IV) was again markedly reduced (*p* < 0.001; Figure 3J). The detailed results of analysis are presented in the supplementary data (Table S3).

#### 2.1.4. Bone Turnover Markers

**Figure 4:**
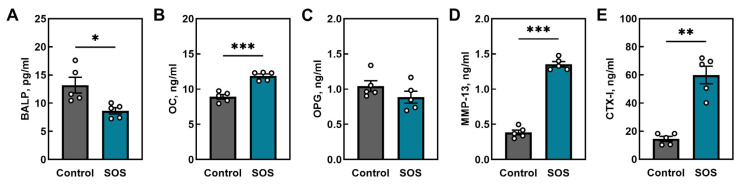
Circulating serum markers of bone turnover in control and SOS pigs. (**A**) Bone-specific alkaline phosphatase (BALP), (**B**) osteocalcin (OC), (**C**) osteoprotegerin (OPG), (**D**) matrix metallopeptidase 13 (MMP-13), and (**E**) C-terminal telopeptide of type I collagen (CTX-I). Data are mean ± SEM with individual values shown (n = 5 per group). Statistical significance: * *p* < 0.05, ** *p* < 0.01, *** *p* < 0.001.

Circulating serum markers of bone turnover were markedly altered in SOS pigs (Figure 4). Bone-specific alkaline phosphatase (BALP), a marker of osteoblastic activity, was significantly reduced in the SOS group (*p* < 0.05; Figure 4A), whereas osteocalcin (OC), another bone formation marker, was significantly elevated (*p* < 0.001; Figure 4B). Serum osteoprotegerin (OPG) did not differ between groups (Figure 4C). In contrast, levels of markers of bone matrix degradation, matrix metallopeptidase 13 (MMP-13) and C-terminal telopeptide of type I collagen (CTX-I), were markedly elevated in SOS pigs (*p* < 0.001 and *p* < 0.01, respectively; Figure 4D,E). The detailed results of analysis are presented in supplementary data (Table S4).

### 2.2. Muscle Analysis

#### 2.2.1. Muscle Fiber Density, Composition, and Diameters

**Figure 5:**
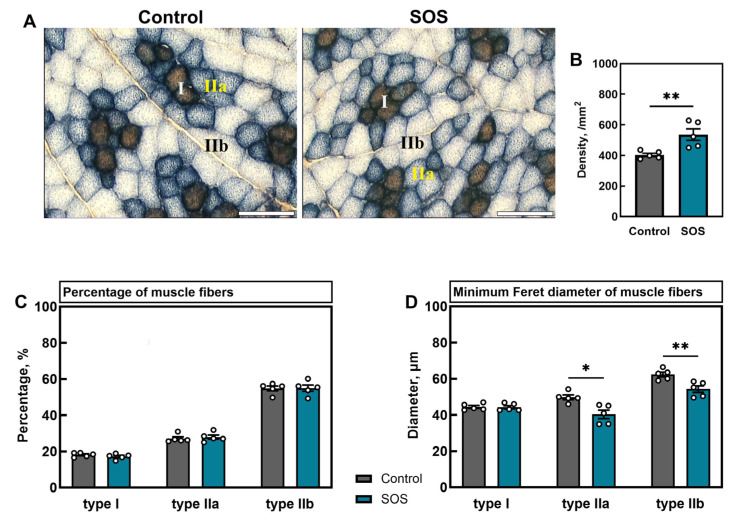
Muscle fiber density, composition, and diameters in control and SOS pigs. (**A**) Representative NADH-TR- and MyHC-stained sections showing type I, IIa, and IIb fibers. Quantification of (**B**) total fiber density, (**C**) percentage of each fiber type, and (**D**) muscle fiber minimum Feret diameter for types I, IIa, and IIb. All the scale bars show 100 μm. Data are mean ± SEM with individual values shown (n = 5 per group). Statistical significance: * *p* < 0.05, ** *p* < 0.01.

Quadriceps femoris muscle cross-sections from SOS pigs revealed hallmarks of secondary sarcopenia. Figure 5A shows representative images of muscle fiber types (I, IIa, and IIb). Total fiber density was significantly increased in SOS animals (*p* < 0.01; Figure 5B). Although fiber-type distribution remained unchanged, with similar percentages of type I, IIa, and IIb fibers in both groups (Figure 5C), the reduction in overall fiber caliber was attributable to a selective decrease in mean diameter of fast-twitch type IIa and type IIb fibers in SOS pigs (*p* < 0.05 and *p* < 0.01, respectively; Figure 5D). The detailed results of analysis are presented in the supplementary data (Table S5).

#### 2.2.2. Gene Expression of Antioxidant Enzymes and Apoptotic Regulators

**Figure 6:**
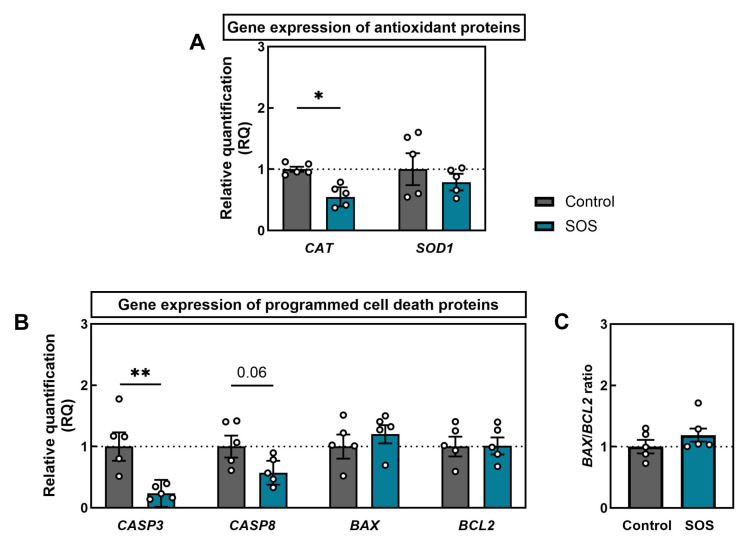
Relative quantification (RQ) of (**A**) antioxidant enzymes *CAT* and *SOD1* mRNA and (**B**) apoptotic regulators *CASP3*, *CASP8*, *BAX*, and *BCL2*. (**C**) *BAX*/*BCL2* mRNA ratio. Data are geomean ± GSE (geometric mean with geometric standard error) with individual values shown (n = 5 per group). Statistical significance: * *p* < 0.05, ** *p* < 0.01; trend: *p* = 0.06.

Expression profiling of antioxidant and apoptotic genes in quadriceps revealed selective dysregulation in SOS pigs (Figure 6). At the antioxidant level, *CAT* mRNA was significantly downregulated in SOS muscle (*p* < 0.05), whereas *SOD1* mRNA expression remained unchanged (Figure 6A). Examination of programmed cell death mediators showed a marked reduction in *CASP3* transcripts in SOS animals (*p* < 0.01) and a trend toward lower *CASP8* mRNA expression (*p* = 0.06) (Figure 6B). In contrast, mRNA levels of pro-apoptotic *BAX* and anti-apoptotic *BCL2* did not differ between groups, resulting in an unaltered mRNA *BAX*/*BCL2* ratio (Figure 6C). The detailed results of the analysis are presented in the supplementary data (Table S6).

## 3. Discussion

Chronic pancreatitis is characterized by persistent inflammation that induces morphological remodeling, frequently culminating in permanent exocrine insufficiency, endocrine dysfunction, and hormonal imbalances. Loss of exocrine function precipitates steatorrhea, nutrient malabsorption, and fat-soluble vitamin deficiencies, contributing to SOS pathogenesis in CP patients [21,22]. Clinical studies report sarcopenia in 17–62% of CP patients [13,23], with up to 70% exhibiting bone disorders [24], and approximately 25% suffering from osteoporosis [18]. In pancreatic cancer, SOS occurs in 19% of patients, while osteoporosis prevalence (52%) exceeds sarcopenia (36%) [25], supporting the concept that BMD reductions may precede sarcopenia development [26]. Recent epidemiological work revealed abnormal BMD in 50–60% of adult pancreatic cancer patients, with osteoporosis in 20% and potential sarcopenia in 48% [27]. Similarly, osteopathic changes occur in 56% of CP patients, with 17% meeting osteoporosis criteria and 39% meeting osteopenia criteria [28]. Research findings on CP in young patients are very limited; however, they indicate similar prevalence rates for sarcopenia and bone loss, regardless of the age of patients [29,30]. Bone fractures were reported in 19% of young CP patients.

Clinical SOS studies in chronic pancreatitis have focused largely on older populations, and animal models have not addressed concomitant bone and muscle effects. The present investigation uniquely employs a juvenile porcine model to comprehensively characterize CP’s skeletal and muscular sequelae. Through combined densitometric, mechanical, and histological bone analyses, alongside serum biomarker profiling, muscle histomorphometry, and gene expression studies, it provides first evidence that cerulein-induced pancreatitis impairs both bone quality and muscle integrity in growing individuals.

Pigs are a relevant preclinical model in many studies [31], including skeletal research due to bone formation and remodeling dynamics closely mirroring humans, with similar fracture patterns from comparable locomotor anatomy [32]. The reported mean age of CP diagnosis in adolescent patients is 9–10 years, with the youngest cases occurring as early as 3–4 years [29,30]. In pigs, peak bone mass is reached at 2–3 years; thus, 4–5-month-old piglets, which, taking into account skeletal system maturation, correspond to approximately 7 years in humans, remain in a predominantly osteogenic phase with active bone formation. This is analogous to teenagers who have not yet reached peak bone mass, which aligns with modeling adolescent onset CP. This enables investigation of growth-related SOS rather than age-related bone loss, making observed pathological changes more translatable to young human populations than smaller laboratory species [32]. However, functional muscle assessment in pigs remains limited due to lack of porcine-specific calibration for CT or MRI imaging methods, or validated human devices such as tensiomyography or MyotonPRO [33,34].

Although exocrine pancreatic function was preserved (no changes in lipase, trypsin, or amylase activities were observed), small intestinal inflammation impaired nutrient absorption and reduced weight gain, thereby accounting for the lower body mass in SOS pigs [19].

Bone densitometry is the primary clinical method for evaluating metabolic bone disorders. Although cortical bone remodels more slowly than trabecular bone [35], cerulein-induced pancreatic inflammation produced a significant decrease in femoral BMD and BMC after just 42 days of chronic disease. Low BMD is a diagnostic criterion for both osteoporosis and osteosarcopenia and is commonly reported in adolescents with CP [29,30]. However, as there are no Z-score standards for pigs, osteoporotic changes in bone homeostasis should be confirmed by additional measurements, such as mechanical testing, histology, or bone turnover markers.

Although CP reduced body weight, femoral mass and length remained unchanged. However, the disease altered mid-diaphyseal geometry; external diameter in the craniocaudal plane, both internal diameters, and CSMI were all significantly reduced. During normal growth, increases in body mass drive medullary cavity expansion via bone modeling; thus, reduced weight gain in SOS animals manifested as impaired diaphyseal widening rather than stunted longitudinal growth. To our knowledge, there are no published DXA or μCT data on femoral geometry in adolescents with CP or SOS, so we cannot relate to this finding. However, this pattern parallels findings in other species, where body mass influences shaft geometry of major load-bearing bones more than bone length [36,37]. These geometric alterations, which shift bone mass closer to the neutral axis, indicate delayed geometric maturation and diminished bone strength in SOS pigs, confirmed by bending tests and consistent with biomechanical principles [36,38].

Among the analyzed femoral mechanical parameters, CP impaired bone elastic properties, evidenced by reductions in yield load and elastic work. Beyond mineral content, bone elasticity is influenced by the organic fraction, primarily collagen structures. The size, organization, and ductility of collagen fibers, along with collagen cross-link density, critically influence elastic behavior [39]. These results suggest that CP may accelerate collagen degradation in bone, a topic revisited when discussing bone turnover marker alterations.

Fracture load and work to fracture remained unchanged in SOS pigs. It should be noted, however, that bone loading does not produce permanent macroscopic changes and plastic deformation but instead initiates microcrack formation. Prolonged or repetitive loading within the plastic region can lead to microdamage accumulation, ultimately resulting in stress (fatigue) fractures, lesions commonly observed in both primary and secondary osteoporosis in young patients [40].

Histomorphometric analysis of trabecular bone in SOS pigs revealed classical osteoporotic alterations; trabecular separation (Tb.Sp) increased, leading to a lower bone volume fraction (BV/TV). These changes reflect impaired osteoblastic activity and diminished bone formation. A trend toward decreased trabecular number (Tb.N) further undermines the structural “framework”, heightening fracture risk. Additionally, a reduction in fractal dimension, indicating increased porosity and loss of architectural complexity [41], parallels observations of increased bone resorption in cortical mid-diaphyseal bone. Together, these findings confirm a compromised trabecular network and increased risk of damage or fractures in physis, consistent with the predominance of trabecular bone loss in early-onset osteoporosis [42].

In the growth plate, changes were observed in the width of all its zones. The reduction in the width of zone I (resting) and zone II (proliferative) suggests impaired chondrocyte proliferation and division, while the reduction in zone IV (ossification) may hinder mineralization of nascent trabeculae [43]. Conversely, hypertrophic zone III was expanded, a change typically reflecting delayed chondrocyte apoptosis and dysregulated hypertrophy, phenomena often driven by chronic inflammation or hormonal imbalance [44]. Indeed, SOS pigs displayed elevated systemic levels of IL-1β, IL-6, and TNFα [19], and in children and adolescents, chronic inflammation characterized by increased cytokine levels can dysregulate the GH/IGF-1 axis, directly impairing growth plate function [45,46]. Although femoral length did not differ between groups, these growth plate alterations may presage longitudinal growth impairment in continuously growing individuals, as commonly observed in chronic inflammatory conditions.

In the present study, changes in serum concentrations of BALP, OC, and CTX-I, established markers of bone formation and resorption in both humans and pigs [47,48], were observed. BALP, whose level was higher in SOS individuals, serves as a sensitive and specific marker of osteoclasts activity [49]. In young individuals, elevated BALP is usually due to increased enzyme activity in growing bone tissue, related to calcium deposition and intensive ossification [50]. Thus, increased BALP concentration directly corresponds with the observed higher BMC and BMD levels in the control animals.

In SOS pigs, the seemingly unexpected rise in OC alongside reduced BALP indicates defective mineralization, not enhanced bone formation. While BALP indicates active osteoblast function and matrix synthesis, OC, produced by osteoblasts, can accumulate in serum when mineralization is defective or in cases of rapid bone loss, as seen in osteomalacia and primary osteoporosis [51,52]. Osteoporosis leads to a decrease in the formation of hydroxyapatite crystals, and, as a result, serum osteocalcin levels increase [53]. Additionally, OC is also found in hypertrophic chondrocytes of the growth plate, with expression levels increasing as chondrocytes progress through hypertrophy [54]. The observed expansion of the growth plate hypertrophic zone in SOS pigs further supports this dual mechanism, where both impaired mineralization and increased chondrocyte-derived OC contribute to elevated serum levels. Similarly, MMP-13, a key enzyme highly expressed in the hypertrophic zone of the growth plate that plays a crucial role in endochondral ossification and programmed cell death of hypertrophic chondrocytes [55], was also significantly increased in SOS pigs. This finding indicates that chondrocyte apoptosis was delayed. Beyond increased MMP-13, which degrades type II and I collagen and other matrix proteins facilitating remodeling, a marked increase in CTX-I, a primary marker of type I collagen degradation, was found in the SOS group. This increase indicates enhanced bone proteolysis by osteoclasts. The increased breakdown of collagen fibers in SOS pigs confirms the observed decreased bone elasticity demonstrated by mechanical testing. Despite evidence of increased osteoclast activity (elevated CTX-I), no effect of induced CP on serum OPG levels was observed. OPG, acting as a “decoy receptor” for RANKL (Receptor Activator for Nuclear Factor κ B Ligand), prevents its binding to receptor RANK, thereby halting osteoclast maturation and proliferation [49]. The lack of OPG level changes usually suggests stable remodeling balance but may also indicate ineffective repair during chronic bone degeneration in a high-bone-turnover osteopenic state with inadequate osteoclast inhibition [56]. This finding underscores the severity of the metabolic disturbance in bone: the body is unable to counter the excess bone loss through OPG-mediated inhibition of osteoclasts.

Quadriceps femoris muscles of SOS pigs exhibited classic sarcopenic changes. Histologically, the overall fiber-type distribution remained unchanged, indicating that no overt fiber type switching or selective loss occurred. However, fiber density was elevated in SOS muscle, reflecting a reduction in fiber caliber rather than true hyperplasia. In particular, fast-twitch type IIa and IIb fibers showed significant atrophy, as evidenced by their diminished size. Such selective type II fiber atrophy is a well-recognized hallmark of sarcopenia and is strongly associated with loss of muscle strength and power [57]. In older adults, preferential wasting of glycolytic type II fibers is a key factor underlying weakness [57], and these findings suggest that a similar fiber-type-specific vulnerability occurs in SOS. Thus, the muscle morphology in SOS pigs clearly points to sarcopenia characterized by type II fiber atrophy, consistent with the expected impact of chronic inflammatory stress on muscle tissue.

Muscles from SOS pigs exhibited selective dysregulation of redox-regulating genes, indicative of increased oxidative stress. Catalase mRNA was significantly downregulated, suggesting an impaired capacity to detoxify hydrogen peroxide (H_2_O_2_) and leaving muscle fibers vulnerable to ROS-mediated damage, typical of sarcopenia [58]. Similarly, Sullivan-Gunn et al. [59] demonstrated that sarcopenia onset in mice coincides with elevated H_2_O_2_ and reduced catalase activity, implicating antioxidant dysfunction in muscle loss. In contrast, SOD1 expression remained stable in SOS muscle, indicating that superoxide dismutation pathways were preserved. This specific impairment in H_2_O_2_ handling may exacerbate protein oxidation, mitochondrial dysfunction, and activation of proteolytic and atrophic signaling pathways in sarcopenic muscle [60].

Connected with changes in oxidative stress markers, a specific pattern in genes governing apoptotic pathways in SOS muscle was observed—a broad downregulation. The mRNA level of *CASP3*, a key executioner of apoptosis and an enzyme involved in proteolytic steps of muscle atrophy, was significantly lower in the quadriceps of SOS pigs. A similar trend was noted for *CASP8* mRNA, an initiator of extrinsic apoptotic signaling, which was marginally reduced in SOS pigs. Meanwhile, transcripts of *BAX* and *BCL2*, pro- and anti-apoptotic regulators of the intrinsic mitochondrial pathway, and their *BAX*/*BCL2* ratio were not altered. This gene expression profile suggests that classical apoptosis was not upregulated in the sarcopenic muscle of this model.

Typically, in catabolic or atrophic conditions, apoptotic signaling increases. For instance, activation of caspase-3 is known to trigger accelerated muscle proteolysis in catabolic muscle wasting [61], and apoptosis has been implicated as one mechanism of muscle fiber loss in age-related sarcopenia [62]. Thus, observed downregulation of *CASP3* expression in SOS muscle is counterintuitive, but it aligns with a potential protective/adaptive response to prevent excessive cell death under chronic stress [63]. Additionally, in young individuals, the intracellular mechanisms of atrophy rely on caspase-independent pathways [64]. The young muscles might be attempting to limit apoptosis in the face of prolonged inflammatory insult, possibly to preserve fiber number. The unchanged *BAX*/*BCL2* ratio further indicates no shift toward pro-apoptotic balance in the mitochondrial pathway [65].

Taken together, these results imply that muscle atrophy in SOS pigs was not driven by widespread apoptosis of fibers. Instead, muscle wasting likely proceeded via caspase-independent pathways—primarily through ubiquitin–proteasome system (UPS) degradation and autophagy-mediated muscle protein breakdown [64,66,67]. The UPS, particularly through muscle-specific E3 ubiquitin ligases atrogin-1 and MuRF1, represents the predominant protein degradation pathway in chronic muscle atrophy, including inflammation-induced muscle wasting [68,69]. Autophagy serves as a complementary proteolytic mechanism, with both pathways working in concert during chronic muscle atrophy [70]. These systems remove contractile proteins and organelles, leading to fiber shrinkage without necessitating fiber death [71]. This mechanism is consistent with age-related sarcopenia studies demonstrating that reduced fiber size, rather than fiber loss, accounts for the bulk of atrophy [72]. The net effect observed in SOS pigs, muscle fiber shrinkage without substantial fiber dropout, aligns with the morphological data and supports this caspase-independent atrophy paradigm [66]. This suggests that chronic-disease sarcopenia relies more on sustained proteostatic imbalance than on classical apoptosis-driven muscle cell loss.

The structural and molecular changes in SOS pig muscles carry important functional implications, especially when considered alongside the concurrent bone findings. The selective atrophy of fast-twitch type II fibers in the quadriceps is expected to impair muscle performance; type II fibers are the primary drivers of high-force, rapid contractions, so their reduced diameter would translate into diminished strength and power output [57]. Although direct muscle strength measurements are not feasible in this pig model, presented results strongly indicate that the SOS pigs had weaker quadriceps muscles than healthy controls.

Functionally, weaker quadriceps muscles mean lower tension generated during locomotion and weight-bearing activities. This reduction in muscle force has direct consequences for the skeleton; according to Frost’s “mechanostat” theory, muscle contractions provide critical mechanical stimuli that drive bone maintenance and growth [73]. In osteosarcopenia, when muscle forces diminish, the bone experiences “unloading”, leading to a shift in bone remodeling toward resorption rather than formation [74]. In growing animals, adequate mechanical loading is essential for achieving PBM. Even partial skeletal unloading can significantly impair bone accrual and strength [75]. Consistent with this, the SOS pigs showed not only muscle atrophy but also deteriorated femoral bone BMD and mechanical properties, and increased collagen breakdown. This indicates that the muscle and bone deficits in this model did not occur in isolation but rather as a coupled phenomenon.

Several limitations of this pilot study should be acknowledged—first, the sample size, which may limit generalizability despite the detection of clear group differences. Second, no direct measurements of UPS (such as MuRF1) and autophagy (such as LC3) pathways were measured directly. Third, the inference of muscle proteostatic imbalance requires validation in larger cohorts and across additional functional bone–muscle groups to confirm its broader applicability. Fourth, because no validated muscle strength protocols currently exist for pigs, the inclusion of surrogate functional measures (such as physical activity monitoring) would strengthen the assessment of overall musculoskeletal function.

Despite these limitations, this investigation offers distinct strengths. It represents the first characterization of SOS in cerulein-induced CP using a juvenile porcine model, yielding novel insights into early-onset SOS. Bone quality was rigorously evaluated using established DXA and three-point bending protocols, and OC, CTX-I, and OPG were employed as recommended markers of bone formation and resorption in both porcine and human studies. In the absence of validated porcine muscle strength assays, muscle alterations were captured through combined fiber-type differentiation and diameter measurements. Finally, classical parametric and nonparametric tests were supported with robust permutation tests and bootstrap resampling to confirm statistical findings via distribution-free methods and mitigate underpowered comparisons.

## 4. Materials and Methods

### 4.1. Animals and Treatment Groups

Experimental procedures have been described in detail previously [19]. Briefly, ten healthy, uncastrated male Polish pbz pigs (*Sus domesticus*), 9–10 weeks of age, were randomly assigned to one of two groups (n = 5 per group)—(i) control and (ii) SOS—in which chronic pancreatitis was induced by cerulein. Animals in the SOS group were administered i.m. injections of cerulein (C2389; Sigma-Aldrich Merck KGaA, Darmstadt, Germany) dissolved in 0.9% saline at a dose of 1 µg/kg body weight once daily for six consecutive days, with 24 h intervals between doses. Control pigs received equivalent volumes of the saline vehicle. Following induction, all pigs were maintained for an additional six weeks on an age-appropriate, nutritionally complete diet. Approved by the Local Ethics Committee, this study was set to run for ten weeks, or until weekly measurements showed a significant drop in body mass in the SOS group versus controls [19]. This threshold was reached on day 42 (week 6), so the experiment ended one week later. On day 49, animals were euthanized under deep anesthesia by sequential i.m. administration of ketamine (350 mg/100 kg b.w.; Biowet, Puławy, Poland), xylazine (200 mg/100 kg b.w.; Sedazin, Biowet), and azaperone (30 mg/100 kg b.w.; Stresnil, Elanco GmbH, Cuxhaven, Germany), followed by an i.v. overdose of pentobarbital (Morbital 26.7 mg/mL; Biowet) at 0.3–0.6 mL/kg b.w.

### 4.2. Sample Collection

Blood was sampled immediately prior to euthanasia and allowed to clot at room temperature before centrifugation (1300× *g*, 10 min, 18 °C). Serum was aliquoted and stored at −86 °C until analysis. Within 5–10 min of euthanasia, biopsy specimens of the quadriceps femoris (*vastus lateralis*) were harvested from the mid-belly of the right limb. Samples destined for mRNA analysis were submerged in StayRNA reagent (#038; A&A Biotechnology, Gdynia, Poland) and stored at −20 °C until RNA extraction. Adjacent portions for histology were trimmed into ~1 cm^3^ blocks with fibers aligned longitudinally, flash-frozen in pre-chilled isopentane (Sigma-Aldrich Merck KGaA, Darmstadt, Germany), and kept over dry ice until storage at −86 °C. Both femora were excised and defleshed, and left femora were individually sealed in zip-lock bags and frozen at −20 °C until analysis. For histology, the distal ends of the right femur were transversely sectioned (including epiphysis and metaphysis) with an MBS 240/E diamond band saw (Proxxon GmbH, Foehren, Germany) and were fixed in 4% (*v*/*v*) neutral-buffered formalin (pH 7.0). Samples were coded upon collection so that all subsequent measurements were performed in a blinded fashion.

### 4.3. Bone Densitometry, Osteometry, and Mechanical Testing

Bone mineral content (BMC) and bone mineral density (BMD) of the left femur were measured by dual-energy X-ray absorptiometry (DXA) using a Lunar iDXA densitometer (GE Healthcare, Madison, WI, USA) with enCORE^®^ software (ver. 7.0, GE Healthcare). Prior to scanning, the system was calibrated with Lunar-compatible bone phantoms of known BMD (0.6–1.4 g/cm^2^). Femora were positioned in the frontal plane and scanned in air without any soft-tissue surrogate; the entire bone was analyzed.

Following densitometry, femora were weighed and measured for length on a digital precision balance and caliper, and the Seedor index (bone weight/length) was calculated. Mechanical integrity was assessed by quasi-static three-point bending on a ZwickRoell 005 universal testing machine (ZwickRoell GmbH & Co., Ulm, Germany) using a 64 mm support span and a crosshead speed of 10 mm/min. Load–deformation curves were processed in Origin (v.2022b; OriginLab, Northampton, MA, USA) to extract biomechanical parameters in the elastic region (yield load, elastic work, stiffness) and plastic region (fracture load, work to fracture) [76]. After mechanical testing, transverse sections of the mid-diaphysis were cut with a diamond band saw, and external and internal diameters were measured in the mediolateral (M–L) and craniocaudal (C–C) planes using a digital caliper. From these dimensions, the cortical index and the cross-sectional moment of inertia (CSMI) were calculated [77].

### 4.4. Bone Histomorphometry

After fixation in formalin bone specimens were decalcified in Decalcifier II (Leica, Wetzlar, Germany) and embedded in paraffin, 4-µm sections (spaced 20 µm) were stained with picrosirius red (PSR) for histomorphometric evaluation of both the growth plate and trabecular bone. Sections were imaged on an Olympus CX43 light microscope (Olympus, Tokyo, Japan). For growth plate analysis, brightfield images were used to measure the thickness of the resting, proliferative, hypertrophic, and calcification zones at least ten locations per section, across two sections per animal. Trabecular bone microarchitecture of distal metaphysis (starting 5 mm away from the growth plate, 10 mm thick), was assessed under polarized light. Quantified parameters included bone volume fraction (BV/TV), trabecular number (Tb.N), trabecular thickness (Tb.Th), trabecular separation (Tb.Sp), and fractal dimension. Two sections per animal were analyzed, with four randomly selected trabecular regions measured per section. All histomorphometric measurements were performed in Fiji (ver. 2.15.0; NIH, Bethesda, MD, USA) [78].

### 4.5. Bone Turnover Markers

Serum concentrations of bone turnover markers were quantified using porcine-specific ELISA kits—bone-specific alkaline phosphatase (BALP; QY-E40206, Qayee Biotech, Shanghai, China), osteocalcin (OC; QY-E40100, Qayee Biotech), osteoprotegerin (OPG; ELK5799, Elk Biotech, Wuhan, China), and matrix metalloproteinase-13 (MMP-13; RK03352, ABclonal, Wuhan, China). Assays were conducted following the manufacturers’ protocols on a Benchmark Plus microplate spectrophotometer (Bio-Rad, Hercules, CA, USA). Each sample was measured in triplicate, and all assays exhibited intra-assay CVs below 8%.

### 4.6. Muscle Histomorphometry

Muscle specimens were mounted on a cryostat chuck using a Tissue Freezing Medium (Leica Biosystems, Nussloch, Germany) and sectioned at −20 °C into 10 µm-thick transverse slices. To distinguish fiber types I, IIa, and IIb, a modified, porcine-validated combined protocol [79,80], incorporating nicotinamide adenine dinucleotide tetrazolium reductase (NADH-TR) activity, followed by immunohistochemical labeling of slow myosin heavy chain on the same section, was employed as previously described [20]. This approach yielded three identifiable fiber populations: brown–blue granulated (type I), blue granulated (type IIa), and unstained (type IIb). For each section, at least 300 fibers from a minimum of 10 distinct muscle bundles were measured to determine fiber minimum Feret diameter [81]. The relative proportion of each fiber type was calculated per bundle, and overall fiber density (fibers/mm^2^) was derived from counts across ten randomly selected fields. All measurements were performed on a Nikon E600 light microscope (Nikon, Tokyo, Japan) and quantified using MultiScan Image Analysis Software (v. 14.02; Computer Scanning Systems Ltd., Warsaw, Poland).

### 4.7. RT-qPCR Analysis of mRNA Expression of Antioxidant Enzymes and Apoptotic Regulators

Muscle samples were homogenized in TRI Reagent (T9424; Sigma-Aldrich Merck KGaA, Darmstadt, Germany) using an Ultra-Turrax T25 dispenser (IKA Werke GmbH and Co., Staufen, Germany), and total RNA was isolated according to the manufacturer’s protocol. RNA yield and purity were assessed spectrophotometrically (NanoDrop Lite; Thermo Fisher Scientific, Wilmington, DE, USA), and integrity was confirmed by electrophoresis on a 2% agarose gel.

Two micrograms of the total RNA were reverse-transcribed into cDNA using High-Capacity RNA-to-cDNA kit (4368814, Applied Biosystems, Walthman, MA, USA). Quantitative qPCR was performed to quantify transcripts encoding antioxidant enzymes (catalase—*CAT* and superoxide dismutase—*SOD1*) and apoptotic regulators (caspase-3—*CASP3*, caspase-8—*CASP8,* apoptosis regulator Bax—*BAX*, and apoptosis regulator Bcl-2—*BLC2*), with glyceraldehyde 3-phosphate dehydrogenase *(GAPDH)* as the endogenous control. Primer sequences were as follows [20]:

*CAT*: forward primer 5′-ATGTGCAGGCTGGATCTCAC-3′ and reverse primer 5′-GCACAGGAGAATCTTGCATC-3′ (product size 155 bp; GenBank XM_021081498.1);

*SOD1*: forward primer 5′-CGAGCTGAAGGGAGAGAAGA-3′ and reverse primer 5′-ACATTGCCCAGGTCTCCAA-3′ (product size 199 bp; GenBank NM_0 01190422.1);

*CASP3*: forward primer 5′-CAAGTTTCTTCAGAGGGGACTGC-3′ and reverse primer 5′-TCGCCAGGAATAGTAACCAGGTGC-3′ (product size 202 bp; GenBank NM_214131);

*CASP8*: forward primer 5′-TCCCAGGATTTGCCTC-3′ and reverse primer 5′-AAGCCAGGTCATCACTGTC-3′ (product size 112 bp; GenBank NM_001031779);

*BAX*: forward primer 5′-TGCCTCAGGATGCATCTACC-3′ and reverse primer 5′-AAGTAGAAAAGCGCGACCA-3′ (product size 199 bp; GenBank XM_003127290);

*BCL2*: forward primer 5′-AGGGCATTCAGTGACCTGAC-3′ and reverse primer 5′-CGATCCGACTCACCAATAC-3′ (product size 193 bp; GenBank NM_214285);

*GAPDH*: forward primer 5′-ATCCCGCCAACATCAAAT-3′ and reverse primer 5′-TCACGCCCATCACAAACA-3′ (product size 165 bp; GenBank XM_021091114.1).

All reactions were run in triplicate, with no-template controls included to exclude contamination. Relative gene expression was calculated using the ΔΔCT method, and the *BAX*/*BCL2* mRNA ratio was determined as an apoptosis index [65].

### 4.8. Statistical Analysis

All statistical analyses were performed in R (version 4.3.0, R Core Team, Vienna, Austria). The experimental unit was the individual pig (n = 5 per group).

Data normality was assessed by the Shapiro–Wilk test. Between-group comparisons employed either Welch’s corrected unpaired *t*-test (for normally distributed data) or the Mann–Whitney U test (for non-normally distributed data).

Given the relatively small sample sizes, classical tests were supplemented with exact permutation tests (oneway_test function, coin package, permutations = 252), which provide distribution-free, exact *p*-values by evaluating all possible rearrangements of the data. Mean confidence intervals (CIs) were calculated using both standard formulae (95% CI) and bootstrap methods (95% CI_boot_) via bias-corrected and accelerated (BCa) resampling (boot function, boot package; replications = 1000). Effect sizes were expressed as Hedges’ g, which corrects Cohen’s d for small-sample bias (cohen.d function, effsize package).

For muscle-fiber composition (percentages of types I, IIa, and IIb), data underwent centered log-ratio (CLR) transformation to address compositional constraints. Between-group differences were tested by permutational multivariate analysis of variance (PERMANOVA; adonis2 function, vegan package; permutations = 999) using Euclidean distance. Individual fiber-type comparisons were further analyzed by calculating means, CIs, Hedges’s g, and applying Wilcoxon rank-sum tests with Benjamini–Hochberg false discovery rate correction.

Gene expression data (RQ values) were log2-transformed to ΔΔCt values for analysis. Between-group differences followed the same statistical approach (Welch’s *t*-test or Mann–Whitney U test supplemented with permutation tests), with confidence intervals and Hedges’s g calculated for geometric means of RQ values.

Statistical significance was set at *p* < 0.05 (two-tailed), with 0.05 ≤ *p* < 0.10 interpreted as a trend. Complete statistical results, including means/geometric means with classical and bootstrap confidence intervals, effect sizes with confidence intervals, and exact *p*-values from parametric/non-parametric and permutation tests, are provided in Supplementary Tables S1–S6.

Graphs were generated using GraphPad Prism (version 10.5.0; GraphPad Software, San Diego, CA, USA). Data are presented as mean ± standard error (SEM), except for mRNA expression, which is reported as geometric mean ± geometric standard error (GSE) due to the exponential nature of ΔΔCt quantification. Statistical annotations in figures reflect results from Welch’s *t*-test or the Mann–Whitney U test, as appropriate.

## 5. Conclusions

The parallel manifestation of bone loss and muscle wasting in pigs which cerulein-induced CP aligns with the concept of SOS. Findings of this study exemplify this link in a secondary (disease-induced) context; CP in young pigs led to a combination of femoral osteopenia and quadriceps sarcopenia. Not only does muscle atrophy likely exacerbate bone loss via reduced mechanical loading, but the disease state and systemic factors (e.g., inflammatory cytokines or endocrine changes) may synchronously affect both muscle and bone metabolism. Conversely, weaker bones and potential microdamage could limit the pigs’ mobility or physical activity, further compounding muscle disuse—a cycle often noted in osteosarcopenic patients. The net result is a functional impairment of the whole musculoskeletal unit—muscles that are weaker and fatigue-prone coupled with bones that are less dense and more fracture-prone.

These data underscore the importance of viewing muscle and bone as an integrated system. In practical terms, interventions aimed at mitigating SOS should address both components; for instance, therapies that reduce chronic inflammation or oxidative stress might simultaneously benefit muscle fibers and bone cells.

In conclusion, the SOS pig model demonstrates that chronic inflammatory disease can induce a coupled degeneration of muscle and bone, thereby providing a valuable insight into how muscle loss can go hand-in-hand with compromised skeletal health in young growing individuals. This highlights the need for an integrated musculoskeletal approach in managing chronic diseases that affect mobility and growth.

## Data Availability

The original contributions presented in this study are included in the article and supplementary data. Further inquiries can be directed to the corresponding author.

## Supplementary Materials

The following supporting information can be downloaded at https://www.mdpi.com/article/10.3390/ijms26167690/s1.

## Abbreviations

The following abbreviations are used in this manuscript:

b.w.	Body weight
BALP	Bone-specific alkaline phosphatase
BAX	apoptosis regulator BAX, BCL-2-like protein 4
BCL2	apoptosis regulator BCL2, B-cell lymphoma 2
BMC	Bone mineral content
BMD	Bone mineral density
BV/TV	Bone volume fraction
CASP3	Caspase-3
CASP8	Caspase-8
CAT	Catalase
CI	Confidence interval
CP	Chronic pancreatitis
CSMI	Cross-sectional moment of inertia
CTX-I	C-terminal telopeptide of type I collagen
GAPDH	Glyceraldehyde 3-phosphate dehydrogenase
GSE	Geometric standard error
MMP-13	Matrix metallopeptidase 13
OC	Osteocalcin
OPG	Osteoprotegerin
PBM	Peak bone mass
RANK	Receptor activator for nuclear factor κ B
RANKL	Receptor activator for nuclear factor κ B ligand
ROS	Reactive oxygen species
RQ	Relative quantification
SEM	Standard error of the mean
SOD1	Superoxide dismutase-1
SOS	Secondary osteosarcopenia
Tb.N	Trabecular number
Tb.Sp	Trabecular separation
Tb.Th	Trabecular thickness
UPS	Ubiquitin–proteasome system
