# Supplementary material for: Coupled Bone–Muscle Degeneration in Chronic Pancreatitis: A Juvenile Porcine Model of Secondary Osteosarcopenia
Source: Int J Mol Sci. 2025 Aug 8;26(16):7690. doi: 10.3390/ijms26167690 (PMC12386997; doi:10.3390/ijms26167690)
Supplement: Supplementary file 1 [file ijms-26-07690-s001.zip › ijms-3802605-supplementary.pdf]

Coupled Bone–Muscle Degeneration in Chronic Pancreatitis: A Juvenile Porcine Model of Secondary Osteosarcopenia

Siemowit Muszyński, Michał Świetlicki, Dorota Wojtysiak, Agnieszka Grzegorzewska, Piotr Dobrowolski, Małgorzata Świątkiewicz, Marcin B. Arciszewski, Iwona Puzio, Joanna Bonior, Agnieszka Tomczyk-Warunek, Maria Mielnik-Błaszczak and Ewa Tomaszewska

Table S1. Detailed results of femoral morphometry and densitometry in control and SOS pigs: means, confidence intervals, effect sizes, and significance tests

Trait	**Control**			**SOS**			**Effect size**		***p*-value**	
	**Mean**	**95% CI[1]**	**95% CI_{boot}[2]**	**Mean**	**95% CI[1]**	**95% CI_{boot}[2]**	**Hedges' g[3]**	**95% CI**	**t-test[4]**	**perm. test[5]**
Bone weight, g	228	221-235	224-233	222	206-239	212-233	−0.52	−1.86-0.92	0.399	0.390
Bone length, mm	179	173-184	175-182	182	1760188	178-185	−0.62	−1.97-0.73	0.307	0.341
Seedor index, g/mm	1.28	1.21-1.35	1.23-1.32	1.22	1.17-1.28	1.19-1.26	−0.99	−1.26-0.41	0.124	0.103
BMD, g/cm^2	0.819	0.770-0.869	0.788-0.855	0.693	0.655-0.731	0.671-0.718	3.23	1.23-5.23	<0.001	0.008
BMC, g	48.52	44.34-52.70	45.84-50.9	43,68	41.32-44.05	41.50-43.40	2.11	0.47-3.75	0.015	0.008
External M-L diameter, mm	21.42	19.81-23.04	20.43-22.41	20.40	19.50-21.30	19.80-21.00	−0.88	−3.36-0.50	0.173	0.206
External C-C diameter, mm	22.64	21.44-23.83	21.89-23.38	20.76	20.19-21.33	20.43-21.12	2.25	0.56-3.93	0.005	0.008
Internal M-L diameter, mm	13.42	12.22-14.63	12.66-14.16	11.73	10.78-12.68	11.18-12.46	1.74	0.20-3.29	0.003	0.024
Internal C-C diameter, mm	14.20	13.33-15.07	12.02-12.88	12.44	11.74-13-14	12.02-12.88	2.51	0.75-4.27	0.002	0.008
Cortical index, %	37.39	35.02-39.56	35.99-38.84	41.25	37.20-45.31	38.34-43.64	−1.36	0.11-2.82	0.053	0.048
CSMI, cm^4	1.04	0.80-1.28	0.89-1.19	0.79	0.67-0.90	0.63-0.88	1.51	0.02-3.00	0.040	0.024

[1] 95% CI: classical 95% confidence interval.
[2] 95% CI_{boot}: 95% confidence interval calculated by bootstrap resampling (Bias-Corrected and accelerated method, BCa).
[3] Hedges' g: standardized mean difference with 95% CI.
[4] t-test: Welch's t-test (or nonparametric equivalent of Mann-Whitney U test where appropriate).
[5] perm. test: exact permutation test.

Table S2. Detailed results of mechanical properties of femora in control and SOS pigs: means, confidence intervals, effect sizes, and significance tests

Trait	Control			SOS			Effect size		*p*-value	
	**Mean**	**95% CI[1]**	**95% CI$_{boot}$[2]**	**Mean**	**95% CI[1]**	**95% CI$_{boot}$[2]**	**Hedges' g[3]**	**95% CI**	**t-test[4]**	**perm. test[5]**
Yield load, kN	2.09	1.83-2.34	1.95-2.29	1.73	1.39-2.08	1.36-1.87	−1.30	−2.75-0.15	0.030	0.032
Elastic work, J	2.26	1.58-3.94	1.91-2.77	1.44	0.97-1.91	1.16-1.73	1.58	−3.09-0.07	0.027	0.024
Stiffness, N	0.98	0.90-1.06	0.93-1.03	1.12	0.86-1.38	0.97-1.30	−0.85	−2.22-0.53	0.200	0.206
Fracture load, kN	2.45	2.17-2.73	2.26-2.61	2.42	2.06-2.78	2.19-2.62	0.09	−1.23-1.40	0.886	0.897
Work to fracture, J	4.17	3.56-4.78	3.72-4.49	3.80	3.35-4.35	3.46-4.03	9.76	−0.60-2.13	0.222	0.230

[1] 95% CI: classical 95% confidence interval.

[2] 95% CI$_{boot}$: 95% confidence interval calculated by bootstrap resampling (Bias-Corrected and accelerated method, BCa).

[3] Hedges' g: standardized mean difference with 95% CI.

[4] t-test: Welch's t-test (or nonparametric equivalent of Mann-Whitney U test where appropriate).

[5] perm. test: exact permutation test.

Table S3. Detailed results of trabecular bone microarchitecture and growth-plate morphology in control and SOS pigs: means, confidence intervals, effect sizes, and significance tests

Trait	Control			SOS			Effect size		*p*-value	
	**Mean**	**95% CI[1]**	**95% CI$_{boot}$[2]**	**Mean**	**95% CI[1]**	**95% CI$_{boot}$[2]**	**Hedges' g[3]**	**95% CI**	**t-test[4]**	**perm. test[5]**
Trabecular bone										
BV/TV, %	28.15	24.60-31.70	26.03-30.47	21.83	18.35-25.30	20.07-24.47	2.02	0.40-3.64	0.008	0.016
Tb.N, /mm	5.11	4.04-6.19	4.35-5.73	4.08	3.40-4.65	3.61-4.47	1.29	−0.15-2.74	0.059	0.071
Tb.Th, µm	57.45	47.92-66.97	51.90-64.07	53.89	49.38-58.33	51.23-56.70	0.54	−0.80-188	0.382	0.389
Tb.Sp, µm	171	133-210	149-198	218	183-253	197-241	−1.42	−2.89-0.06	0.038	0.048
Fractal dimension, --	1.43	1.37-1.49	1.39-1.47	1.37	1.32-1.41	1.34-1.40	1.24	−0.20-2.68	0.064	0.071
Growth plate zones										
Resting zone, µm	147	132-163	137-156	112	98-126	102-119	2.73	0.90-4.56	0.001	0.008
Proliferative zone, µm	336	300-372	314-358	272	233-311	251-302	1.92	0.33-3.51	0.010	0.023
Hypertrophic zone, µm	160	1330187	147-183	233	185-280	196-258	−2.10	−3.75-−0.46	0.009	0.016
Calcification zone, µm	425	369-481	374-450	272	254-291	263-287	4.11	1.78-6.43	<0.001	0.008

[1] 95% CI: classical 95% confidence interval.

[2] 95% CI$_{boot}$: 95% confidence interval calculated by bootstrap resampling (Bias-Corrected and accelerated method, BCa).

[3] Hedges' g: standardized mean difference with 95% CI.
[4] t-test: Welch's t-test (or nonparametric equivalent of Mann-Whitney U test where appropriate).
[5] perm. test: exact permutation test.

Table S4. Detailed results of bone turnover markers in control and SOS pigs: means, confidence intervals, effect sizes, and significance tests

Trait	Control			SOS			Effect size		*p*-value	
	**Mean**	**95% CI[1]**	**95% CI$_{boot}$[2]**	**Mean**	**95% CI[1]**	**95% CI$_{boot}$[2]**	**Hedges' g[3]**	**95% CI**	**t-test[4]**	**perm. test[5]**
BALP, pg/ml	13.21	9.31-17.11	10.99-15.97	8.65	7.10-10.19	7.66-9.55	1.72	0.18-3.27	0.028	0.008
OC, ng/ml	8.91	8.01-9.80	8.27-9.40	11.88	11.12-12.76	11.38-12.32	−4.00	−6.29-−1.172	<0.001	0.008
OPG, ng/ml	1.04	0.83-1.26	0.94-1.25	0.89	0.65-1.12	0.75-1.07	0.77	−0.60-2.14	0.215	0.222
MMP-13, ng/ml	0.384	0.288-0.480	0.330-0.450	1.35	1.15-1.46	1.29-1.43	−10.75	−15.92-−5.57	<0.001	0.008
CTX-I, ng/ml	14.52	9.56-19-47	11.26-17.11	59.99	42.70-77.29	46.42-69.69	−4.00	−6.29-−1.172	0.001	0.008

[1] 95% CI: classical 95% confidence interval.
[2] 95% CI$_{boot}$: 95% confidence interval calculated by bootstrap resampling (Bias-Corrected and accelerated method, BCa).
[3] Hedges's g: standardized mean difference with 95% CI.
[4] t-test: Welch's t-test (or nonparametric equivalent of Mann-Whitney U test where appropriate).
[5] perm. test: exact permutation test.

Table S5. Detailed results of muscle fiber density, composition and diameters in control and SOS pigs: means, confidence intervals, effect sizes, and significance tests

Trait	Control			SOS			Effect size		*p*-value	
	**Mean**	**95% CI[1]**	**95% CI$_{boot}$[2]**	**Mean**	**95% CI[1]**	**95% CI$_{boot}$[2]**	**Hedges' g[3]**	**95% CI**	**PERMANOVA[4] or t-test[5]**	**Bootstrap[6] or perm.test[7]**
Percentage									0.488	0.999
Type I, %	18.14	16.66-19.62	17.06-18.89	17.16	15.30-19.03	15.78-18.13	0.65	−0.70-2.00	0.631	0.301
Type IIa, %	27.08	24.35-29.81	25.95-29.90	27.89	25.75-31.03	26.23-30.28	−0.31	−1.63-1.02	0.631	0.566
Type IIb, %	54.78	51-07-58.49	51.01-56.33	54.95	50.06-59.84	51.42-57.57	−0.04	−1.36-1.27	0.841	0.961
Minimum Feret fiber diameter										
Type I ,μm	44.44	42.20-46.67	43.16-46.06	44.26	41.80-46.73	42.78-45.82	0.08	−1.24-1.40	0.888	0.818

Type IIa ,µm	49.77	46.09-53.45	47.58-52.40	40.33	33.86-46.79	36.14-43.72	2.01	0.40-3.63	0.011	0.008
Type IIb ,µm	62.40	58.76-66.-4	60.14-64.59	54.27	49.21-59-34	50.89-57.01	2.07	0.44-3.70	0.008	0.008
Fibers density, /mm²	404	373-435	385-423	536	432-641	469-603	−1.93	−3.52-−0.33	0.022	0.008

[1] 95% CI: classical 95% confidence interval.
[2] 95% CI_{boot}: 95% confidence interval calculated by bootstrap resampling (Bias-Corrected and accelerated method, BCa).
[3] Hedges' g: standardized mean difference with 95% CI.
[4] PERMANOA or t-test: PERANOVA test (overall effect and statistic and detailed analysis for each fiber type with false discovery rate correction for multiple comparison) for percentage distribution.
[5] t-test: Welch's t-test (or nonparametric equivalent of Mann-Whitney U test where appropriate) for fiber diameters and density.
[7] bootstrap: bootstrap test (Bias-Corrected and accelerated method, BCa) for percentage distribution.
[5] perm. test: exact permutation test for fiber diameters and density.

Table S6. Detailed results of gene expression of antioxidant enzymes and apoptotic regulators muscles in control and SOS pigs: geometric means (geomean), confidence intervals, effect sizes, and significance tests

Trait	**Control**		**SOS**		**Effect size**		***p*-value**	
	**Geomean**	**95% CI_{boot}[1]**	**Geomean**	**95% CI_{boot}[1]**	**Hedges' g[2]**	**95% CI**	**t-test[3]**	**perm. test[4]**
Antioxidant proteins								
CAT	1.000	0.891-1.122	0.549	0.367-0.820	−2.28	−3.81-−0.68	0.012	0.008
SOD1	1.000	0.526-1.899	0.788	0.552-1.124	-0.52	−1.65-−0.65	0.401	0.381
Programmed cell death proteins								
CASP3	1.000	0.562-1.780	0.236	0.136-0.411	−2.86	−4.60-−1.06	0.001	0.008
CASP8	1.000	0.631-1.586	0.574	0.350-0.942	−1.30	−2.55-0.01	0.052	0.064
BAX	1.000	0.610-1.640	1.199	0.816-1.760	0.46	−0.70-1.59	0.446	0.452
BCL2	1.000	0.661-1.513	1.007	0.701-1.448	0.02	−1.01-1.14	0.972	0.976
BAX/BCL2 ratio	1.000	0.748-1.337	1.190	0.898-1.677	0.68	-0.50-1.83	0.266	0.270

[1] 95% CI_{boot}: 95% confidence interval calculated by bootstrap resampling (Bias-Corrected and accelerated method, BCa).
[2] Hedges' g: standardized mean difference with 95% CI.
[3] t-test: Welch's t-test (or nonparametric equivalent of Mann-Whitney U test where appropriate).
[4] perm. test: exact permutation test.
